# PEP-Patch: Electrostatics in Protein–Protein Recognition, Specificity, and Antibody Developability

**DOI:** 10.1021/acs.jcim.3c01490

**Published:** 2023-11-07

**Authors:** Valentin J. Hoerschinger, Franz Waibl, Nancy D. Pomarici, Johannes R. Loeffler, Charlotte M. Deane, Guy Georges, Hubert Kettenberger, Monica L. Fernández-Quintero, Klaus R. Liedl

**Affiliations:** † Department of General, Inorganic and Theoretical Chemistry, and Center for Molecular Biosciences Innsbruck (CMBI), University of Innsbruck, 6020 Innsbruck, Austria; ‡ Department of Statistics, University of Oxford, Oxford OX1 2JD, United Kingdom; § Roche Pharma Research and Early Development, Large Molecule Research, Roche Innovation Center Munich, Penzberg 82377, Germany

## Abstract

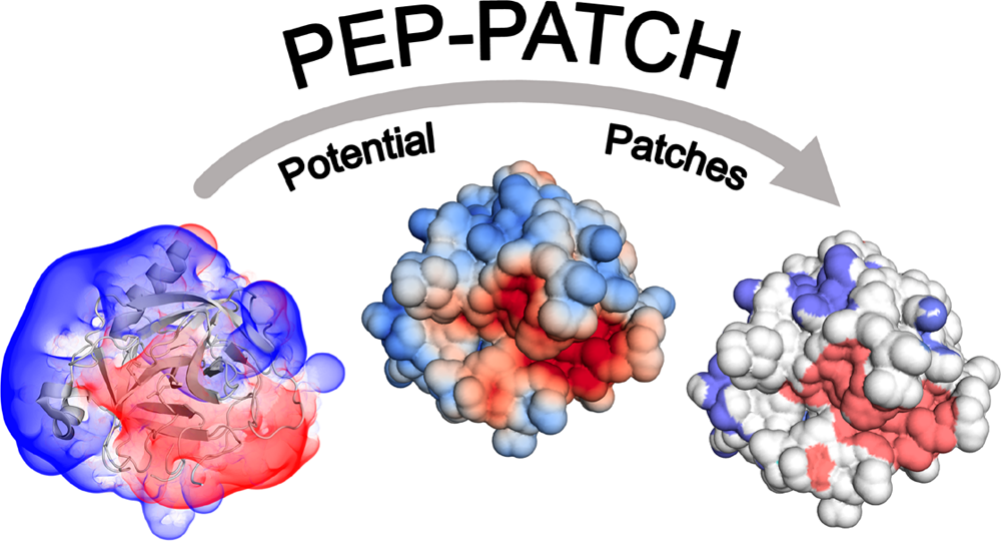

The electrostatic properties of proteins arise from the number and distribution of polar and charged residues. Electrostatic interactions in proteins play a critical role in numerous processes such as molecular recognition, protein solubility, viscosity, and antibody developability. Thus, characterizing and quantifying electrostatic properties of a protein are prerequisites for understanding these processes. Here, we present PEP-Patch, a tool to visualize and quantify the electrostatic potential on the protein surface in terms of surface patches, denoting separated areas of the surface with a common physical property. We highlight its applicability to elucidate protease substrate specificity and antibody–antigen recognition and predict heparin column retention times of antibodies as an indicator of pharmacokinetics.

## Introduction

Electrostatic forces are central in molecular biology, codetermining protein folding, protein–protein interactions, protein–DNA/RNA interactions, ion binding, dimerization, and protein stability.^1−3^ Additionally, they affect the p*K*_a_ values of ionizable groups in proteins and DNA/RNA.^4,5^ As such, they are key to characterizing the biophysical properties of proteins. Due to their long-ranged character, they are especially important to the complex multistep process of molecular recognition. This process requires a balance of entropic and enthalpic components,^6,7^ with varying contributions during the different steps from the unbound to the fully bound state. Electrostatics are a critical component of the enthalpic contributions, dominating and guiding the recognition process, especially when the binding partners are still distant from one another.^8^

Electrostatic forces affect molecular binding through interactions not only between the binding partners but also with the solvent. This is because solvent molecules must be displaced from the binding interface, which introduces a large desolvation penalty that needs to be overcome by an interplay of attractive electrostatic and hydrophobic interactions upon protein–protein or protein–ligand association. Additionally, it has been reported that long-range electrostatic interaction networks increase specificity of proteins while restricting their flexibility.^9^ On the other hand, weak electrostatics can be associated with conformational variability and, consequently, cross-reactivity.^9^ Protein folding and thermal stability are also influenced by electrostatics. In particular, polar interactions are a major contributor to hydrogen bonding, and hydration of charged and polar amino acids has a profound impact on correct protein folding.^1−3^ Due to protonation state changes, pH can influence protein stability and function.^5,10^ Thus, understanding the role of electrostatics in protein function is crucial to advance, guide, and facilitate protein engineering and design.

### Surface Patches

Macromolecular interactions are often mediated by a single dominant interaction surface. In the case of primarily electrostatic interactions, this implies that continuous surface patches with a high charge density are likely candidates for interaction surfaces.^11^ Similar arguments hold for hydrophobic interactions, which might also be mediated by a single hydrophobic patch.^12^

The electrostatic potential around a protein in solution is routinely calculated using Poisson–Boltzmann or Generalized Born calculations.^13−15^ For visualization of the resulting potential,^16^ iso-surfaces can be displayed in standard molecular visualization packages such as PyMOL^17^ or VMD.^18^ However, this visualization is not optimal for quantification of the results since the potential in the first hydration shell, which is an important indicator of interaction strength, is not visible. More informative is the projection of such a potential onto the protein surface, as defined, e.g., by the solvent-accessible surface.

On the other hand, when developing quantitative scores of electrostatics or hydrophobicity, surface patches have often been used for example as input features for machine learning models or for the development of descriptors such as the electrostatic surface area.^19−23^

Programs to search for continuous patches have been developed since the 1990s,^24,25^ their molecular visualization was implemented in software such as MOE^26^ and others. While noncommercial solutions are available, they are usually tied to a single use case, focusing mostly on either visualization of the total surface potential or calculation of descriptors.^27−29^ Often these are also available only via a web server, inhibiting easy inclusion into automatized workflows. As such, further development aside from the original purpose or custom tools building upon these are hampered.

### PEP-Patch

Here, we present the Python tool PEP-Patch for the calculation, quantification, and visualization of continuous surface patches. The tool generates a protein surface around a user-provided PDB structure and interpolates the values of the user-provided potential on this surface. Patches are then calculated by searching for continuous areas on this surface where all values are either above (positive patch) or below (negative patch) a certain cutoff. The patches can be visualized in PyMOL, with customization and filtering options available to further adapt the patch calculation to various workflows. While our tool calculates an electrostatic potential using APBS by default, it can be used with any combination of a surface representation and a three-dimensional potential, thus providing a versatile building block for biomolecular analysis.

PEP-Patch is freely available on GitHub (https://github.com/liedllab/surface_analyses) under an MIT license. As such, it provides an open-source foundation for the future development of Python based descriptors and algorithms dealing with molecular surfaces. Here, we provide examples investigating the electrostatic surfaces of proteases and antibodies and show an application to a user-calculated potential map for antibody hydrophobicity.

## Methods

### Electrostatic Potential

PEP-Patch uses the Advanced Poisson–Boltzmann Solver (APBS) software to compute the electrostatic potential using the Poisson–Boltzmann equation.^13,30,31^ A NaCl concentration of 0.1 M is used by default, without titrating residues. PDB2PQR is used to prepare any supplied pdb structure before the APBS calculation, adding missing heavy atoms, guessing the correct protonation state, and introducing hydrogens correspondingly. However, since the electrostatic potential strongly depends on the charges in the system, it is vital that the user carefully checks the choice of protonation states.

### Surface and Patch Generation

Smooth molecular surfaces can be defined by using a Gaussian surface, a solvent-accessible surface, or a Connolly type surface. The potential is then linearly interpolated at every surface vertex. Positive and negative surface patches are defined using an isolevel by searching for connected components above/below this value in the graph defined by the triangulated surface. Similar procedures are commonly used to find protein surface patches.^21^

The output of our tool includes the surface and interpolated values in the numpy storage format (npz), as well as a color-coded surface in ply format for visualization in molecular visualization systems such as PyMol or VMD.^32^ A list of patches can be generated, providing the type of each identified patch, its area, and the residue contributing the most to the patch size. A list of all inputs to the tool to generate the figures in this publication is provided in the SI.

### Quantitative Scores for Electrostatics

We define five different quantitative scores for the electrostatic properties of a molecule as an example of simple descriptors enabled by the implemented surface analysis functions. To do so, we start from the electrostatic potential map obtained from a Poisson–Boltzmann calculation. We then select grid voxels that are solvent accessible within a defined distance cutoff from the protein. By default, this distance cutoff is defined to be ten Å from the protein surface.

The *total* score is defined simply as the integral of the electrostatic potential over that region. In a very simplified view, it can be considered as the interaction strength with a positively charged substance, given that this substance is evenly distributed in the selected volume.

We also define positive and negative scores, which only include contributions of the positive and negative regions in the electrostatic potential. Again, they can be understood as an interaction strength with a charged substance, this time imagining that the substance is distributed only in the respective part of the volume.

Finally, we define *high* and *low* scores, which are defined in the same way as the *positive* and *negative* scores, except that they include only regions above and below a given electrostatic potential cutoff.

## Application and Illustrative Examples

In this study, we present a tool to quantify and characterize the electrostatic surface properties of proteins. Potential applications include the investigation of molecular recognition and the development of descriptors for pharmacokinetics. Here, we apply our tool to three different case studies: substrate specificity of proteases based on their surface properties, molecular recognition upon antibody affinity maturation, and biophysical properties and pharmacokinetics of antibodies.

### Protease Substrate Recognition

The protease recognition process has been shown to be electrostatics driven, where the substrate preferences can be predicted from charge complementarity in the binding interface.^8,33^

They are enzymes that proteolytically cleave peptide bonds and play a key role in a variety of different physiological processes, ranging from complex signaling cascades, blood coagulation, and food digestion to key aspects of the immune system such as programmed cell death and digestions of cells.^34,35^ These very distinct and broad biological functions require vast differences in specificity and promiscuity.^33,36,37^ While some proteases reveal high specificity for substrate sequences, others are more promiscuous, cleaving a variety of different substrates. An example are digestive proteases that cleave food proteins and thus need to function on many different substrates. Substrate specificity of proteases is conveyed by molecular interactions occurring at the protease–substrate interface in the binding cleft of the protease.^33^ Here, we use our tool to compare different proteases based on their electrostatics.

**Figure 1:**
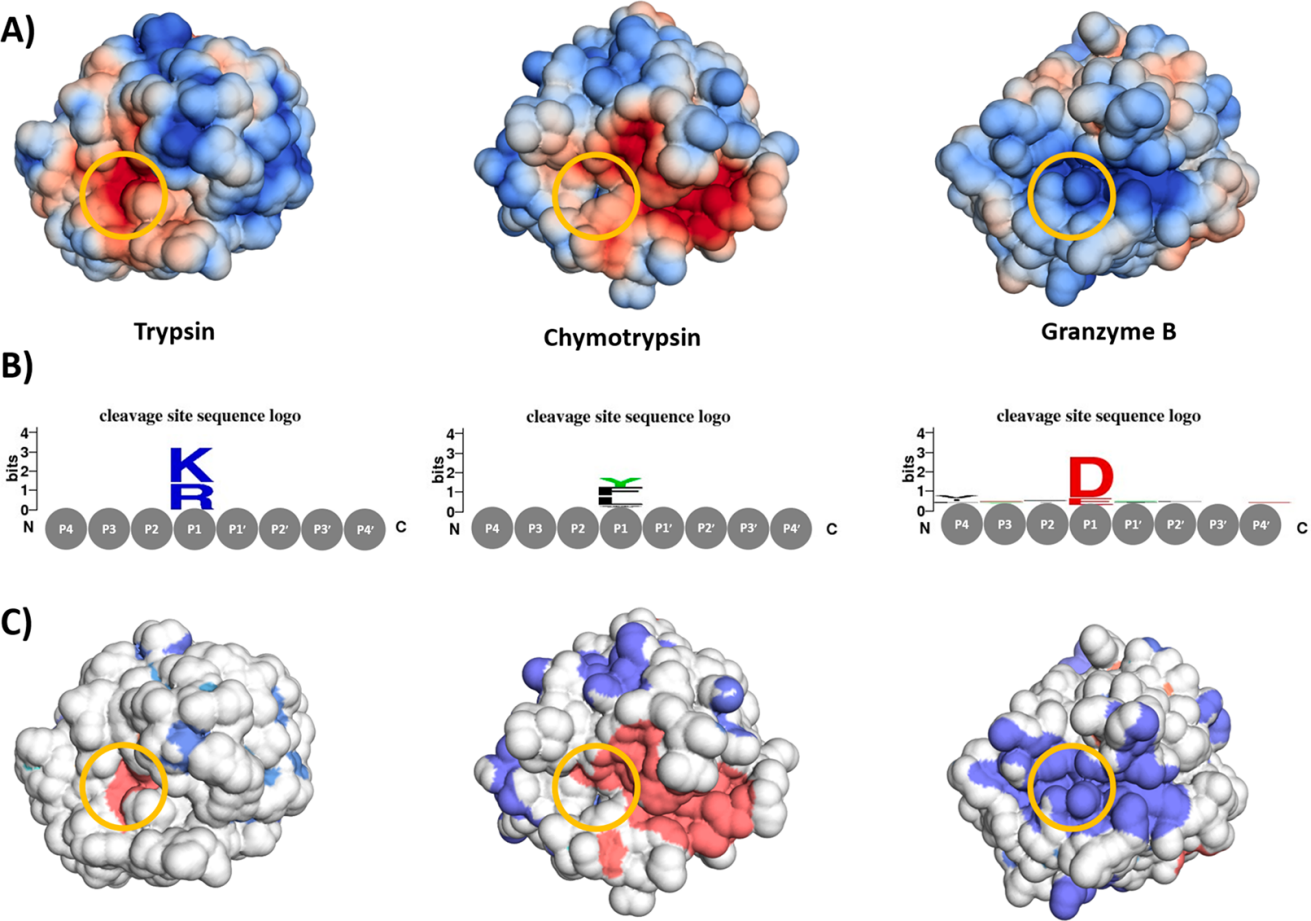
Electrostatic potentials around three proteases differing in their substrate preferences. The location of the active binding site S1 is highlighted with a yellow circle. (A) The electrostatic potential on the solvent-accessible protein surface around Trypsin, Chymotrypsin, and Granzyme B. The magnitude of the electrostatic potential is shown ranging from red for a negative potential to blue for positive potential. (B) Cleavage site sequences logos obtained from the MEROPS database show the most common substrate of the respective protease. (C) Positive (blue) and negative (red) protein surface patches showcase continuous areas with similar electrostatic potential.

Figure 1A shows the comparison of three proteases differing in their substrate preferences. We mapped the electrostatic potential (Figure 1A) based on X-ray structures for all three proteases (PDB accession codes: 1PQ7 for Trypsin, 4CHA for Chymotrypsin, and 1FQ3 for Granzyme B) and show that, by considering the electrostatic potential around each protease binding cleft (red represents negatively charged patches, blue positively charged patches), the substrate preference^38^ can be inferred. Granzyme B shows a preference for negatively charged substrates, which is reflected by the positive patch in the binding site (Figure 1A and C, top left). Trypsin prefers more positively charged substrates, again reflected by a large negative patch, which encompasses the binding site and the area around it (see Figure 1C, bottom right). Chymotrypsin prefers neutral substrates. While its binding cleft is surrounded by a positive patch, the binding site itself is mostly neutral with a small negative patch close to the active site, thereby only allowing neutral residues. The positive and negative protein surface patches are illustrated in Figure 1C, and the residues contributing most to the absolute area ascribed to an electrostatic patch are provided in SI Table 1.

### Antibody–Antigen Recognition

The high therapeutic potential of antibodies in combination with their versatility makes them excellent candidates to study.^39,40^ Here, we focus on the interface of two different antibodies binding to the same chemokine CXCL13 antigen.^41^ Structurally, the antigen-binding fragment of an antibody (Fab) is composed of a heavy and light chain, parts of which form the antigen-binding site, the paratope. The paratope is primarily found within six hypervariable loops, the complementarity determining region (CDR) loops.^39^ In addition to the CDR loops, residues in the framework as well as the relative interdomain orientation between the heavy and light chain can strongly influence antigen recognition.^42−45^ It is well established that, for antibodies, single-point mutations can change the binding site conformations and thereby affect biophysical properties.^46−48^ The antibody variants investigated here, the parental 3B4 and the optimized E10, have substantial differences in affinity and stability resulting from only four point-mutations located in the CDR-L3 loop.^41^ 3BA and E10 differ only for the CDR-L3 loop sequence, which in the former is **S**S**Y**T**RR**DTYV, while in the latter is mutated into **A**S**A**T**LL**DTYV, replacing polar and positively charged residues with neutral ones. These changes result in a 3-fold decrease in *k*_off_ and a 5 °C increase in thermal stability. As the antibodies are otherwise the same, the total electrostatic potential descriptor here mostly describes the change introduced by these point-mutations. Note, however, that the electrostatic potential is to some degree conformation dependent, and a more detailed analysis should take all relevant solution conformers into account.

**Figure 2:**
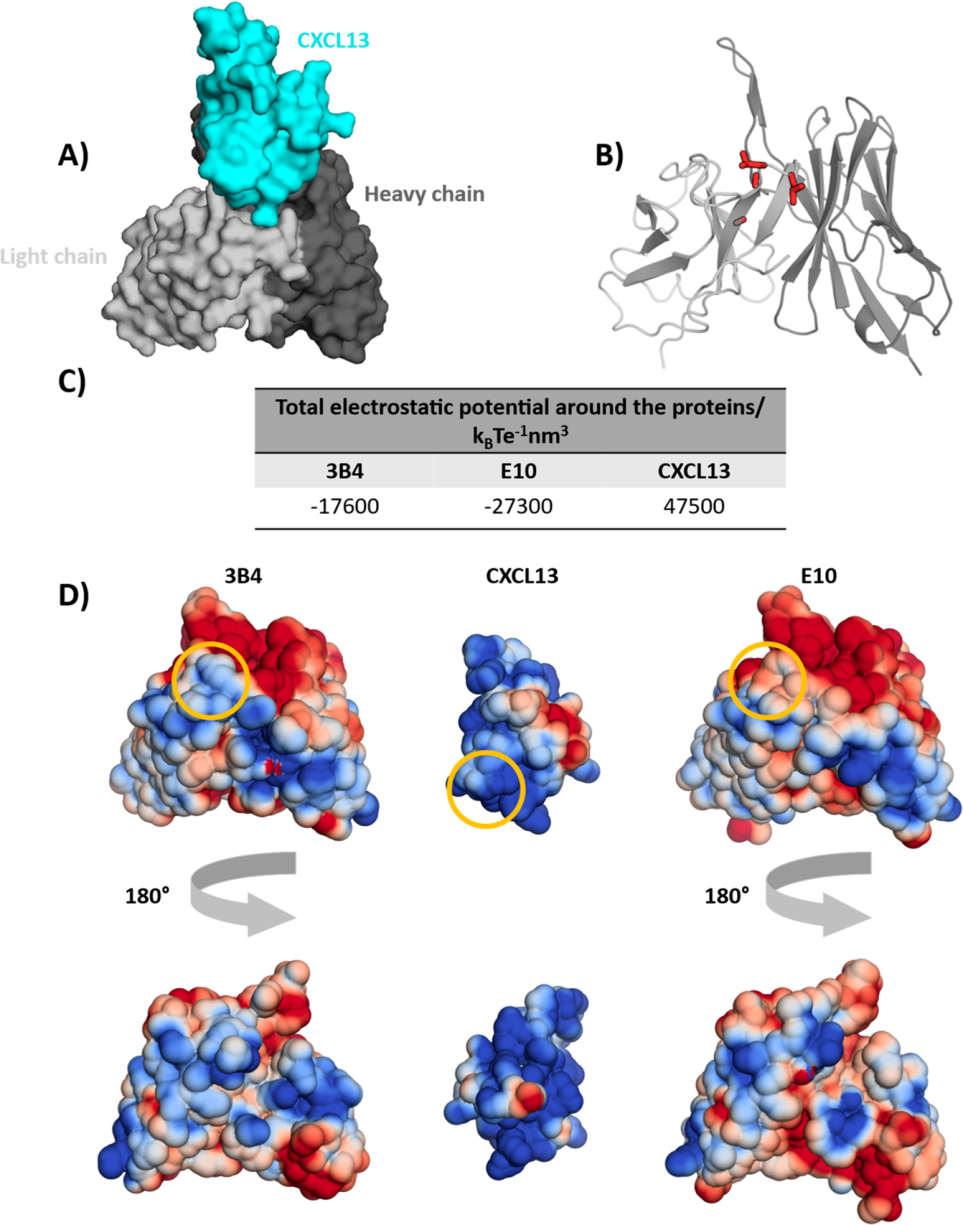
Electrostatic potential in antibody recognition. (A) Antibody–antigen binding mode, derived from the available X-ray structure of the E10 complex (PDB accession code: 5CBE), showing the antibody in gray and the antigen in cyan. (B) Structural representation of E10 with the relevant four CDR-L3 mutations shown in red. (C) Summary of the total electrostatic potential around the proteins, rounded to the nearest hundred. (D) Electrostatic potential mapped to surfaces of antibodies 3B4 and E10 (both sides) and the CXCL13 antigen, ranging from positive potential in blue to negative potential in red. The area surrounding the CDR-L3 mutations and its binding region on the antigen are marked with yellow circles.

To calculate the electrostatic potential, we used crystal structures from the PDB (accession codes: 5CBA for 3B4 and 5CBE for E10). The antigen was separated, and the antibodies were cut to the same sequence length, encompassing Fv only. From comparison of the resulting data in Figure 2, we find that these four point-mutations contribute to an improved electrostatic complementarity of the E10 variant with CXCL13. This is reflected in a decrease in the total calculated electrostatic potential when the four mutations are introduced, thereby improving the fit to the strongly positively charged antigen. This is also visible in the electrostatic potential map, where the respective areas corresponding to the point mutations are comparably more negative. Overall, the observed improved electrostatic complementarity of the antigen to E10 helps to explain the experimentally observed affinity increase.

### Antibody Developability–Predicting Differences in Pharmacokinetics

Another critical aspect in developing antibodies, apart from antibody–antigen recognition, is pharmacokinetics.

Biophysical properties of antibodies, such as surface charges or hydrophobicity and the isoelectric point, are thought to be responsible for changes in pharmacokinetics, efficacy, dose intervals, and application route. Heparin retention chromatography is a common measure of antibodies serum half-life.^49^ Heparin is a negatively charged polysaccharide that resembles the glycocalyx, a saccharide layer on the inside of epithelial cells. Interaction with the glycocalyx is believed to increase the propensity of a compound to be taken up into the cell by pinocytosis, followed by digestion and thus the retention time in heparin chromatography correlates with serum half-life of monoclonal antibodies.^49^

### Antibody Structure Models

We used heparin data from Kraft et al.^49^ for a set of 137 antibodies described in the data set by Jain et al.^50^ Antibody Fv structures were modeled from sequence using the machine learning tools DeepAb^51^ and ImmuneBuilder,^52^ as well as homology models from MOE.^26^ The models were used to elucidate the influence of different antibody conformations on the respective electrostatic potential. The default settings were used during the modeling process for all tools. In addition, for 49 of these 137 antibody sequences,^50^ crystal structures were available and included in the calculation for comparison to the denovo modeling software.

Here, we compare our positive electrostatic potential descriptor to relative heparin column retention times from Kraft et al.^49^ and show that the electrostatic potential of the antibody variable fragments (Fv) is a key determinant for pharmacokinetics, reflected in a compelling correlation with the experiment. Independent of the structure models or X-ray structures used as starting points in our calculations, we find similar correlations. This is a strong indication that the electrostatic potential, due to its long-ranged nature, may be less conformation dependent than other biophysical properties, such as hydrophobicity.

**Figure 3:**
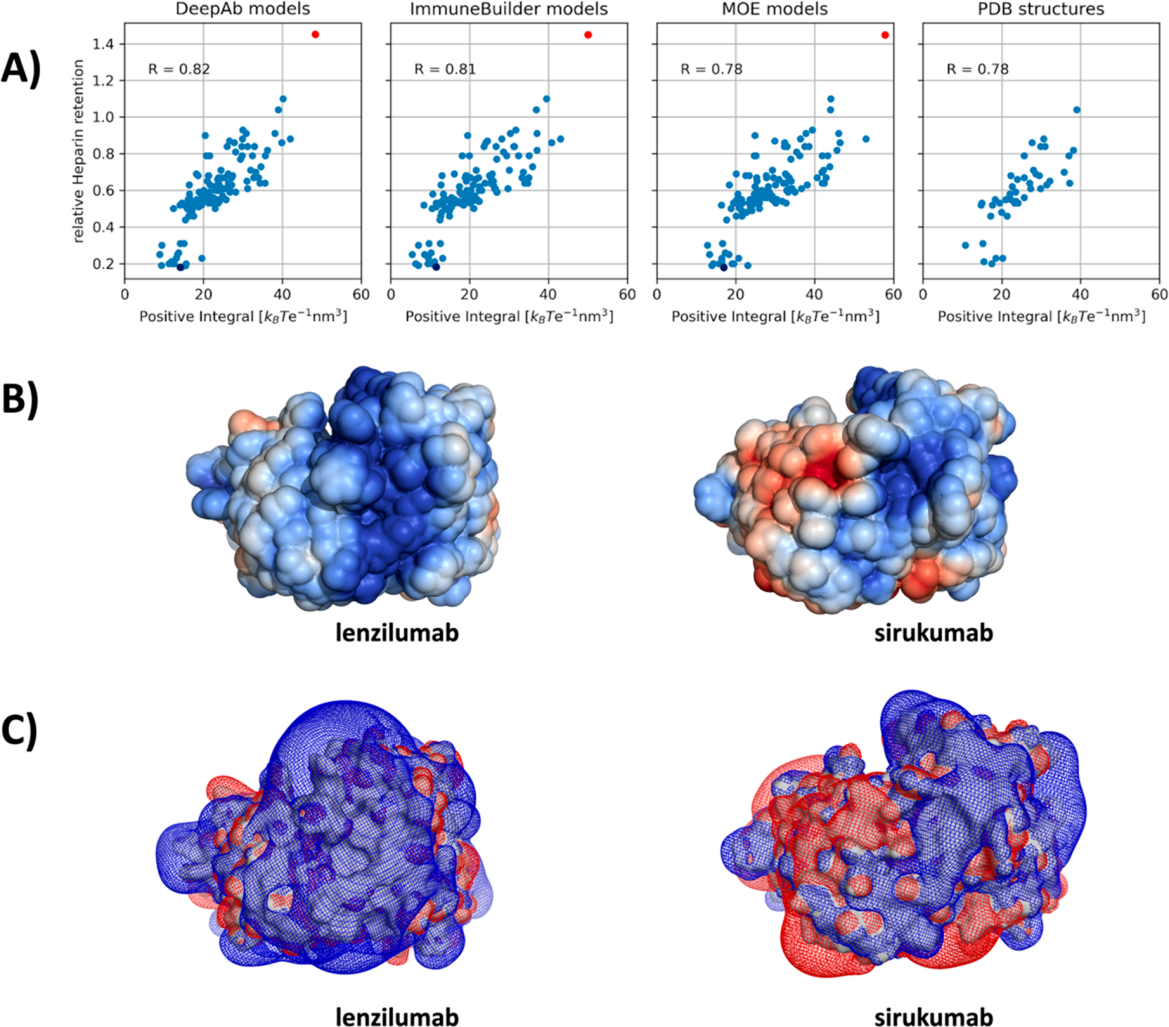
(A) Scatter plot of relative heparin retention time against the integral of the positive electrostatic potential over the solvent-accessible volume, for the investigated 137 antibodies using the DeepAb models, ImmuneBuilder models, MOE models, and the 49 crystal structures from the PDB. (B) Electrostatic potential around the DeepAb models of lenzilumab (depicted as red dot) and the sirukumab (shown as blue dot), which exhibit the highest and lowest heparin retention time, respectively. (C) Mesh isopotential surface around the two DeepAb models of lenzilumab and the sirukumab.

At a low positive electrostatic potential score (below 20 *k*_B_ Te^–1^ nm^3^) there appears to be no correlation with the heparin retention time. This is probably due to very weak interactions with the column. The highlighted points in Figure 3A represent the antibodies with the highest and lowest heparin column retention times. These differences in the experimental retention times are also reflected in the electrostatic potential mapped to the surface; i.e., lenzilumab shows a higher positive electrostatic potential compared with sirukumab, which is substantially more negative (Figure 3B).

To demonstrate the usefulness of our results, we compare our tool to those of other commonly used scores for protein charges. We compute the average net charge of each model using the Protein Properties tool in MOE and plot the resulting values against the same heparin data (Figure S1). Furthermore, we produce an analogous plot using the total area of positive patches calculated with the conformational sampling option turned on in the MOE (Figure S2). While all three tools perform well in general, we note that the correlation between our tool and the heparin retention times is slightly higher. To make it easier for the users to match patch data to protein residues, PEP-patch tool provides the residues that contribute the most area to each patch. Furthermore, if the input structure is an antibody fragment, it can detect which patches contain atoms of the complementarity determining regions (CDRs) using ANARCI.^53^

### Application to Other Potential Maps

**Figure 4:**
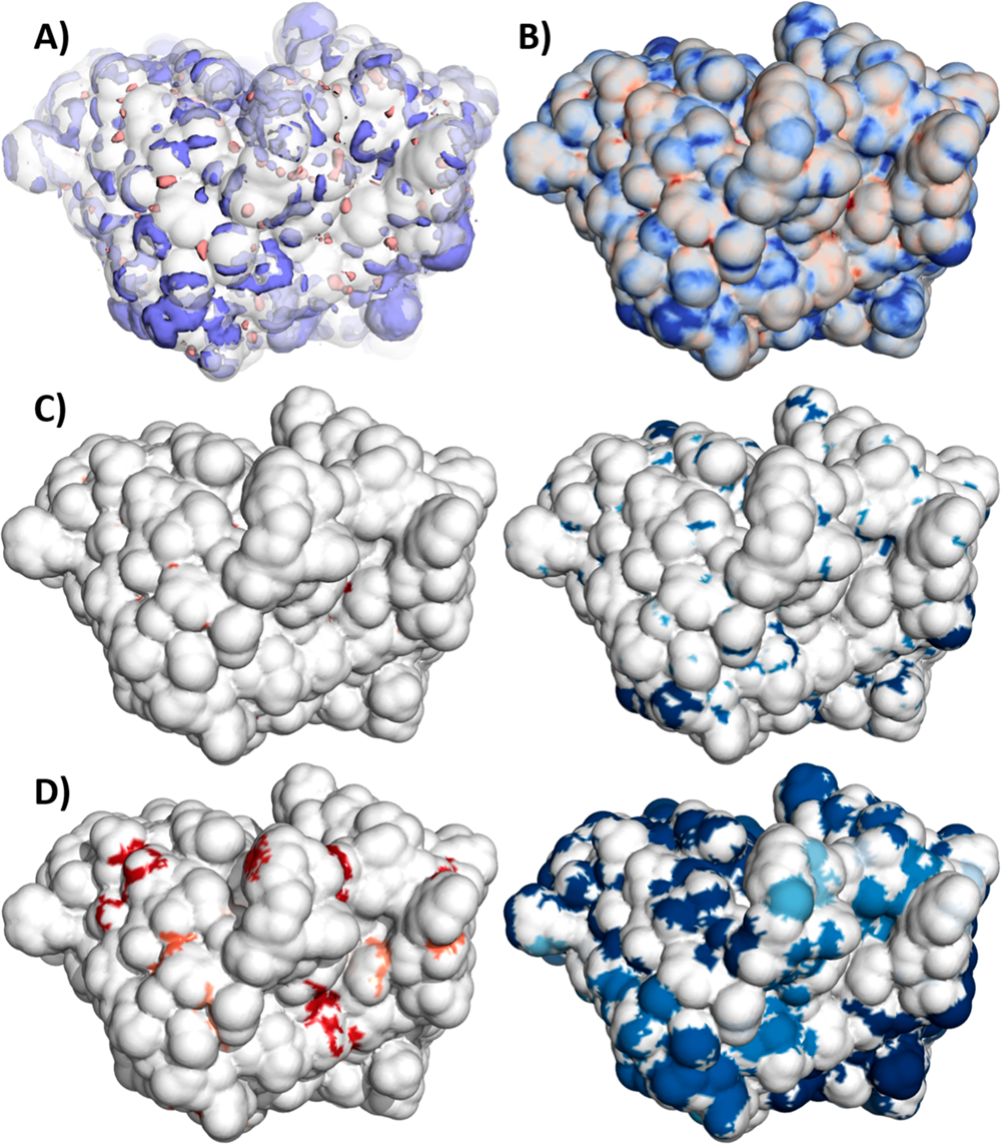
(A) Isosurface at 0.1 kcal mol^–1^ Å^–3^ of GIST free energy of hydration around the Fv solvent-accessible surface. (B) Hydration free energy mapped to solvent-accessible surface, ranging from negative values in blue (hydrophilic) to positive values in red (hydrophobic). (C) Patches based on surface vertices with a free energy of hydration above 0.2 kcal mol^–1^ Å^–3^ (left) or below −0.2 kcal mol^–1^ Å^–3^ (right). Patches were colored according to area, red to white for positive patches and blue to white for negative patches. (D) Patches based on surface vertices with free energy of hydration above 0.02 kcal mol^–1^ Å^–3^ (left) or below −0.02 kcal mol^–1^ Å^–3^ (right). To reduce visual noise introduced by many small patches, a size cutoff of 10 A^2^ was used, where only patches with an area above the cutoff are displayed.

PEP-Patch can be applied to any combination of protein structures and any user-provided potential map. To demonstrate the wide applicability of our tool, we map the localized hydration free energy to an antibody Fv from a grid inhomogeneous solvation theory (GIST)^54−56^ calculation first presented in Waibl et al.^57^ In Figure 4, areas with a negative solvation free energy can be considered hydrophilic, while areas with a positive solvation free energy are hydrophobic. Visualizing and identifying regions of interest in GIST data are often difficult, as the amount of data is often visually overwhelming. By mapping the potential to the solvent-accessible surface, we can directly show the positions where the influence of the free energy of hydration is most pronounced, within the first hydration shell.

Patches were calculated for the free energy of hydration around the paratope of bevacizumab (PDB code 1BJ1). Overall, IgG antibodies can be considered hydrophilic, as they act in serum, thereby necessitating sufficient solubility. As such, we find more patches showing negative free energy in Figure 4C when compared to the very few and small positive patches using the same cutoff. To emphasize hydrophobic areas, a low cutoff was used in Figure 4D, essentially showing all hydrophobic areas. Whereas hydrophobic areas are hard to spot in the total potential mapped to the surface due to their small size, the split into positive and negative patches and the change of cutoff allow an easy identification of both hydrophilic and hydrophobic areas. For rugged densities, such as the free energy on hydration shown here, calculating patches may result in many small patches. For visualization, it is advisible to choose a continuous color map in such cases, as the standard qualitative colormap does not provide enough colors to show all patches. Additionally, patches can be filtered by maximum number and minimum or maximum size, focusing the analysis on only the most key areas of the molecule (see Figure 4D).

## Conclusion

We present the PEP-Patch tool, which calculates and quantifies patches on molecular surfaces. The tool can directly calculate the electrostatic potential of proteins, although other potential maps/3D-grids can be supplied by the user. By splitting the potential into negative and positive contributions, continuous areas with similar biophysical properties are identified, termed surface patches. Additionally, it allows one to directly visualize and quantify the electrostatic potential around different proteins, guiding the design of biotherapeutic proteins. As application examples, we show that the electrostatic potential can explain biomolecular recognition, substrate specificity, and even pharmacokinetics of antibodies. The split into positive and negative patches is helpful in locating areas of interest that might get lost in the total electrostatic potential surface. Furthermore, the tool quantifies the resulting patches and identifies the residues that contribute the most to an electrostatic patch, which can inform rational protein design. PEP-Patch is open source, enabling future developments to build on its surface analysis workflow, patch generating algorithm, and visualization routines.

## Data Availability

The code for the PEP-Patch tool is available on Github under the MIT license https://github.com/liedllab/surface_analyses. The structures used in this manuscript are publicly available, with the PDB codes: 1PQ7, 4CHA, 1FQ3, 5CBE, 5CBA, 1BJ1, and the models are available on Github. All inputs to the PEP-Patch tool necessary to recalculate the presented data can be found in the Supporting Information.

## Supporting Information Available

Input commands to PEP-Patch and additional procedural details for all figures. Relative heparin retention correlated to antibody net charge and positive patch area, both calculated with MOE. Detailed patch results for proteases and antibody–antigen recognition. (PDF)

## Abbreviations

PB: Poisson–Boltzmann
Fab: antigen-binding fragment
CDR: complementarity determining region
GIST: Grid Inhomogeneous Solvation Theory
