# Supplementary material for: PEP-Patch: Electrostatics in Protein–Protein Recognition, Specificity, and Antibody Developability
Source: J Chem Inf Model. 2023 Nov 7;63(22):6964–71. doi: 10.1021/acs.jcim.3c01490 (PMC10685443; doi:10.1021/acs.jcim.3c01490)
Supplement: Supplementary file 1 — ci3c01490_si_001.pdf [file ci3c01490_si_001.pdf]

SUPPORTING INFORMATION

PEP-Patch: Electrostatics in Protein-Protein Recognition, Specificity and Antibody Developability

Valentin J. Hoerschinger[‡1], *Franz Waibl*[‡1], *Nancy D. Pomarici*[‡1], *Johannes R. Loeffler*[1], *Charlotte M. Deane*[2], *Guy Georges*[3], *Hubert Kettenberger*[3], *Monica L. Fernández-Quintero*[*1], *Klaus R. Liedl*[*1]

[1]Department of General, Inorganic and Theoretical Chemistry, and Center for Molecular Biosciences Innsbruck (CMBI), University of Innsbruck, Innsbruck, Austria.

[2]Department of Statistics, University of Oxford, Oxford, UK.

[3]Roche Pharma Research and Early Development, Large Molecule Research, Roche Innovation Center Munich, Penzberg, Germany.

*To whom correspondence should be addressed:

Monica L. Fernández-Quintero: Monica.Fernandez-Quintero@uibk.ac.at

Klaus R. Liedl: Klaus.Liedl@uibk.ac.at

Inputs to PEP-Patch and Additional Method Details

Figure 1 – Proteases

Protein structures were downloaded from the PDB (IDs: 1PQ7, 4CHA, 1FQ3) and solvent, ligand and duplicate chain atoms were removed. The command for e.g., trypsin then was the following, using custom colormaps to color the patches:

pep_patch_electrostatic trypsin.pdb -o trypsin --apbs_dir trypsin --pos_patch_cmap coolwarm --neg_patch_cmap coolwarm_r --ply_out trypsin

Figure 2 – E10 and 3B4 antibodies

Protein structures were downloaded from the PDB (IDs: 5CBA, 5CBE) and solvent, ligand and duplicate chain atoms were removed. The antigen was extracted into a single pdb file. The antibodies were stripped to just their Fv sequence, ensuring the same number of residues in both pdb files. The example input for E10 down below was also run for 3BA and the

antigen, applying a limit to the range of the colormap:

pep_patch_electrostatic E10.pdb -o E10 --apbs_dir E10_apbs --ply_out E10 --ply_clim -2 2

Figure 3 – Antibody Fv models

Antibody Fv models were created from sequence using ImmuneBuilder, DeepAb and MOE. The models were then run through the tool using standard settings:

pep_patch_electrostatic ab.pdb -o ab --apbs_dir ab_apbs --ply_out ab

The resulting surfaces and the grid from the APBS calculation were directly visualized for lenzilumab and sirukumab.

Figure 4 – Solvation Free Energy from GIST

The GIST free energy grid and its corresponding structure were loaded into PEP-Patch, using various cutoffs to emphasize positive and negative patches:

pep_patch_electrostatic gist.pdb gist-A-dens.dx -o gist_normal -c 0.2 -0.2 --pos_patch_cmap Reds_r --neg_patch_cmap Blues_r --ply_cmap coolwarm --ply_out gist_normal

pep_patch_electrostatic gist.pdb gist-A-dens.dx -o gist_low -c 0.02 -0.02 --pos_patch_cmapReds_r --neg_patch_cmap Blues_r --ply_cmap coolwarm --ply_out gist_low

Supplemental Figures and Tables

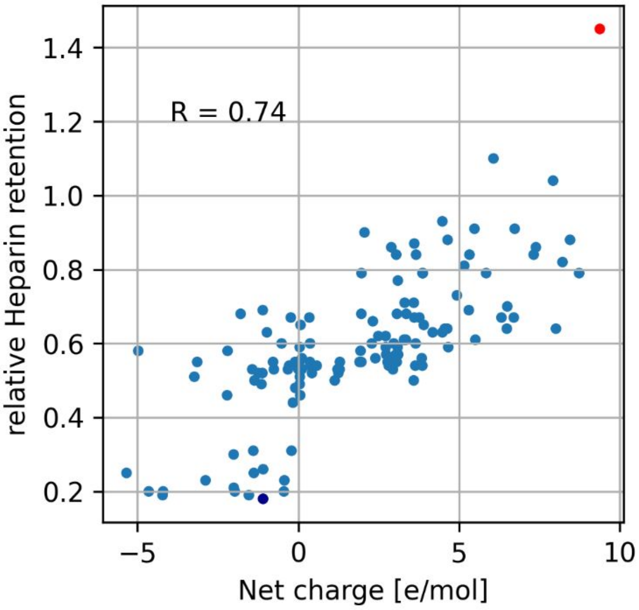

Figure S1: Net charge of the antibodies calculated with MOE, correlated with the relative

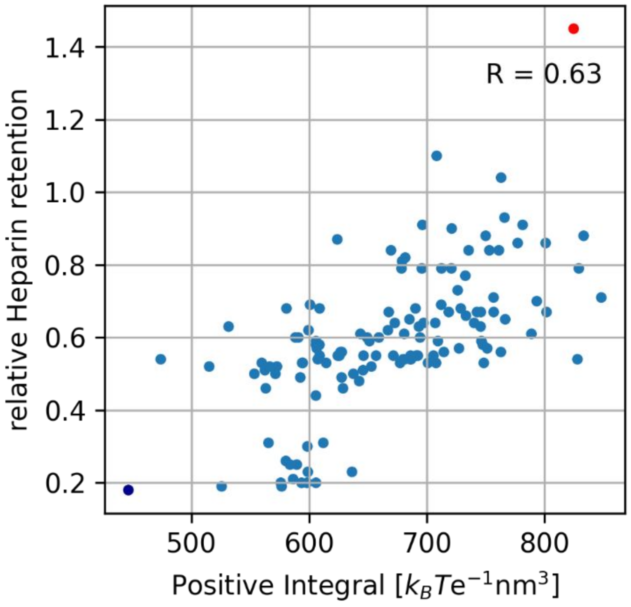

heparin retention time of the antibodies.

Figure S2: Relative heparin retention times correlated with the positive patch area provided by MOE. As in Figure 3 of the main text, lenzilumab and sirukumab are shown as red and blue dots, respectively.

Table S1: Residues contributing the most to the absolute value ascribed to an electrostatic patch for the results presented in Figure 1 and Figure 2.

Trypsin			
type	npoints	area	main_residue
positive	729	117.679463	LYS188
positive	721	116.802802	ARG166
positive	404	65.1777161	ARG88
positive	339	55.5023517	LEU73
positive	314	51.9860782	ARG119
positive	312	51.1179195	PRO91
positive	274	42.6745675	ARG35
positive	233	36.3607999	ARG75
positive	157	27.3872189	SER83
positive	156	24.2751487	ARG69
positive	121	20.8056815	THR76
positive	69	10.5907007	SER147
positive	64	10.2707108	VAL89
positive	45	6.38613449	GLY37
positive	38	6.00178771	GLN63
positive	37	5.95083239	SER123
positive	38	5.21988658	TRP138

positive	15	2.52468883	THR164
positive	13	2.48472098	SER184
positive	16	2.23689742	LEU231
positive	13	2.05111792	ASN151
positive	11	1.64536576	THR171
positive	14	1.2597704	ARG69
positive	6	0.61085037	SER172
positive	5	0.26974551	GLY19
positive	6	0.15791531	GLY37
positive	6	0.12617516	GLY37
positive	10	0.09310352	SER72
positive	6	0.08882306	LYS154
positive	6	0.07800999	SER32
positive	6	0.06634554	ARG119
positive	6	0.01333066	THR156
positive	6	0.00865386	TRP138
positive	6	0.00497744	LYS188
negative	860	138.922415	GLN192
negative	274	45.585308	ASP26
negative	109	17.5881279	ALA239
negative	84	11.7466516	GLY182
negative	64	8.16579672	ASP201
negative	29	5.18963711	SER59
negative	31	4.5760538	SER222
negative	22	1.99737441	TYR221
negative	8	1.22504268	PHE179
negative	7	1.17262072	ASP26
negative	10	0.92894108	ALA239
negative	5	0.89138293	ALA239
negative	5	0.71569338	ALA173
negative	14	0.60340463	VAL224
negative	6	0.1454201	ALA209
negative	6	0.13372595	MET178

negative	6	0.09828762	TYR221
negative	6	0.09431558	ALA209
negative	6	0.06020165	ALA209
negative	6	0.03681595	SER190

Chymotrypsin			
type	npoints	area	main_residue
positive	7943	1312.55305	LYS93
positive	793	129.316283	CYS1
positive	657	106.886058	LYS84
positive	567	89.8772885	LYS202
positive	224	33.8326658	SER190
positive	84	11.061201	SER189
positive	61	9.4477061	ARG145
positive	49	8.27085818	LYS36
positive	38	6.65437407	THR222
positive	42	5.22824885	THR138
positive	27	4.13088373	SER119
positive	30	3.43381332	LEU123
positive	17	2.82974537	LYS203
positive	21	2.76323791	ALA126
positive	14	2.25412412	LYS82
positive	16	2.0558185	GLY140
positive	15	1.86168774	VAL210
positive	7	1.53223932	LYS203
positive	18	1.31616817	VAL238
positive	2	0.40298813	SER45
positive	4	0.35489217	ALA56
positive	2	0.31336404	SER218
positive	6	0.12671322	THR232
positive	6	0.12182391	VAL231
positive	1	0.11814837	LYS90
positive	6	0.1179208	SER190

positive	6	0.06820048	VAL60
positive	6	0.02323131	VAL3
positive	6	0.02227583	GLY226
positive	6	0.01481454	TRP237
positive	6	0.00013212	GLY226
negative	7772	1284.84966	PHE39
negative	217	36.6977191	TYR146
negative	112	18.6612543	ASN245
negative	107	18.3547825	GLU49
negative	21	3.03103765	ASP194
negative	18	2.72215966	ASP35
negative	19	2.53942174	GLY196
negative	13	1.2697544	SER214
negative	10	0.74375484	TRP29
negative	2	0.54043506	SER127
negative	6	0.37061736	ILE85
negative	4	0.27287853	ASP128
negative	6	0.27247725	VAL67
negative	6	0.26031518	GLY25
negative	1	0.16794804	ASP35
negative	6	0.14523811	THR139
negative	6	0.12813773	GLY25
negative	6	0.09596294	SER214
negative	10	0.06544949	THR138
negative	6	0.02181548	VAL23
negative	6	0.00540477	ASP102
negative	6	9.52E-05	LEU33

Granzyme B			
type	npoints	area	main_residue
positive	13528	2537.12056	LYS188
positive	6210	1166.51848	LYS192
positive	1715	319.434124	ARG110

positive	284	52.747294	LYS131
positive	266	49.4404739	ARG27
positive	256	47.4218536	GLN210
positive	172	34.12302	ARG87
positive	181	34.0747797	ARG217
positive	62	12.8301405	GLN37
positive	50	9.00056703	HIS153
positive	48	8.33189323	ALA139
positive	40	7.49171164	LYS149
positive	42	5.35365681	TRP141
positive	25	4.17261904	THR144
positive	8	1.68319615	ASN219
positive	12	1.58704484	GLN129
positive	6	1.50931899	ARG172
positive	15	1.32892487	VAL52
positive	14	1.18532356	ARG41
positive	16	1.09542089	GLY193
positive	4	0.89515589	ASN219
positive	6	0.66125942	GLN156
positive	6	0.39810759	MET224
positive	6	0.39638472	TYR32
positive	5	0.3301109	GLN163
positive	2	0.32290892	TYR245
positive	6	0.25931213	ARG41
positive	6	0.20154489	THR189
positive	1	0.1994544	ARG87
positive	3	0.14950518	PRO120
positive	6	0.10986478	THR144
positive	5	0.10781241	PRO120
positive	6	0.10062683	ALA31
positive	6	0.0899475	VAL52
positive	6	0.06556522	ARG41
positive	6	0.04740378	GLN210

positive	6	0.04277298	VAL158
positive	1	0.03983786	PHE82
positive	6	0.02321586	GLY43
positive	6	0.01928432	ASP49
positive	6	0.01655484	VAL130
positive	6	0.01208086	LEU33
positive	1	0.00983955	PRO146
positive	6	0.00869647	LEU171
positive	6	0.00633369	VAL235
positive	6	0.00511543	LYS188
positive	1	0.00385118	ARG226
positive	6	5.90E-05	LYS239
negative	1941	365.909166	ASP176
negative	773	143.496239	GLU75
negative	191	35.0881447	TYR245
negative	108	19.0411565	GLU186
negative	56	8.16209073	LEU181
negative	35	5.87209033	ASP37
negative	27	4.24344888	TYR245
negative	19	3.86103188	ASP50
negative	16	3.83266122	GLU109
negative	10	1.3100794	PHE51
negative	14	1.16955116	VAL130
negative	4	0.33034444	VAL52
negative	6	0.32864457	ALA112
negative	1	0.25826574	TRP59
negative	5	0.24752879	SER190
negative	1	0.16138442	GLU109
negative	5	0.11500604	ARG114
negative	1	0.07741278	ASP49
negative	1	0.06624385	CYS228
negative	3	0.06390481	ARG114
negative	6	0.0516718	MET242

negative	6	0.03608713	PRO28
negative	6	0.03530829	VAL130
negative	6	0.03212897	TYR245
negative	6	0.02849412	GLN129
negative	6	0.0101261	SER100
negative	6	0.00997878	VAL162
negative	6	0.00726417	GLU75
negative	6	0.0065888	ALA112
negative	6	0.00256342	PRO225

3b4			
type	npoints	area	main_residue
positive	2512	428.789227	LYS62
positive	924	157.953631	LYS13
positive	716	122.127738	LYS45
positive	538	91.4414097	GLN1
positive	364	60.4605843	ARG94
positive	101	16.3860577	LYS23
positive	95	15.4982389	LYS66
positive	55	7.83732517	THR68
positive	44	7.15705111	LYS42
positive	46	6.5778703	ASN52
positive	29	5.35951325	ASN69
positive	38	5.05948052	LYS62
positive	30	4.8667855	SER67
positive	24	4.43226678	SER27
positive	22	4.41414826	HIS60
positive	20	3.06133387	ARG94
positive	26	2.66122019	VAL51
positive	11	1.80785497	ARG94
positive	5	1.3310678	ARG54
positive	9	1.31000165	THR108
positive	8	1.19027251	SER27

positive	5	0.82715504	ARG93
positive	3	0.53213758	SER84
positive	6	0.29187666	GLN1
positive	1	0.27476218	GLY57
positive	1	0.25447908	SER82
positive	2	0.2473777	LYS103
positive	5	0.24251281	SER27
positive	1	0.16593153	LYS12
positive	6	0.14984186	PHE63
positive	1	0.12056577	ALA43
positive	1	0.07442074	PRO52
positive	6	0.05451902	THR68
positive	6	0.023932	ALA71
negative	6259	1064.54774	TYR100
negative	2285	393.890378	GLU108
negative	1007	170.014341	ASP85
negative	662	114.060594	GLU73
negative	547	93.2590157	SER113
negative	150	26.3165134	PRO96
negative	94	17.3531439	ASP72
negative	42	6.88287011	GLN1
negative	40	6.84576262	THR74
negative	29	4.92600511	ASP27
negative	28	4.52016513	PRO15
negative	19	3.67606112	LYS62
negative	17	2.43431612	GLU10
negative	6	0.90466735	GLU10
negative	7	0.61822932	SER30
negative	10	0.48974506	ASP50
negative	10	0.16774348	ASP50
negative	6	0.10199716	TRP32
negative	6	0.0664382	TYR98
negative	6	0.0490952	ASP100

negative	6	0.04611165	SER89
negative	6	0.0220922	TYR91
negative	6	0.01229754	PHE49
negative	6	0.00750903	SER34
negative	6	0.00624069	ASN58
negative	6	5.45E-07	LEU78

e10			
type	npoints	area	main_residue
positive	1268	203.225349	SER30
positive	946	150.861561	LYS62
positive	613	101.975944	GLN1
positive	611	97.6618333	LYS13
positive	594	94.4855458	HIS39
positive	375	59.9213342	ARG83
positive	346	55.8554497	LYS23
positive	184	30.1797673	SER84
positive	96	15.2559337	ARG61
positive	87	13.6223383	ARG54
positive	90	13.2826421	TRP103
positive	53	8.70396899	ARG54
positive	47	7.84472203	SER18
positive	49	7.6503087	LYS23
positive	56	6.7328299	SER84
positive	44	6.44911319	TRP47
positive	31	4.74468231	SER27
positive	29	4.55253409	ARG83
positive	17	2.51942562	THR105
positive	13	2.37319543	SER12
positive	14	2.15572966	THR28
positive	23	2.08518759	ALA13
positive	6	1.05484618	ASN69
positive	6	0.77171049	LEU94

positive	7	0.59753573	TYR79
positive	6	0.46724068	ARG66
positive	3	0.44333242	SER18
positive	5	0.42644175	ILE51
positive	5	0.37793912	LYS19
positive	6	0.30603493	PHE63
positive	1	0.2296781	ASN69
positive	1	0.22690341	SER82
positive	1	0.22336508	LYS62
positive	6	0.19701217	GLY49
positive	10	0.1763086	GLY49
positive	1	0.1558938	SER25
positive	1	0.13864917	SER2
positive	6	0.06627006	TYR32
positive	6	0.06355005	LEU4
positive	6	0.0617888	LYS62
positive	6	0.05808696	GLN1
positive	1	0.05680838	SER82
positive	6	0.05569998	ILE51
positive	1	0.03626656	SER27
positive	6	0.03074973	ILE69
positive	6	0.02795613	TYR36
positive	6	0.01597983	MET48
positive	6	3.34E-05	VAL37
negative	7521	1214.99069	TYR100
negative	3273	526.63814	LEU106
negative	760	122.388021	GLU73
negative	408	68.0369264	GLU46
negative	407	61.8737191	GLN43
negative	279	45.2769291	THR68
negative	206	33.1061826	SER113
negative	155	24.5935023	ASP82
negative	130	20.1110056	GLU81

negative	107	17.180903	GLU10
negative	96	15.0344981	ASP72
negative	69	11.1367083	SER2
negative	54	8.69032998	GLN3
negative	50	7.81803451	VAL51
negative	38	4.79547475	SER25
negative	18	3.19399885	GLN3
negative	21	3.16127543	LEU45
negative	13	2.33366788	ASP27
negative	7	0.85954202	GLN43
negative	6	0.45500448	LEU47
negative	5	0.33013279	SER14
negative	1	0.22811838	ASP100
negative	4	0.19204181	GLY99
negative	5	0.17999254	ALA43
negative	10	0.13938728	ASP95
negative	1	0.1148977	PRO55
negative	1	0.01698894	GLN43
negative	6	0.01377142	VAL58
negative	6	0.01110618	VAL33
negative	6	0.0011787	ILE75
